# Spatio-temporal processes drive fine-scale genetic structure in an otherwise panmictic seabird population

**DOI:** 10.1038/s41598-020-77517-w

**Published:** 2020-11-26

**Authors:** Lucy J. H. Garrett, Julia P. Myatt, Jon P. Sadler, Deborah A. Dawson, Helen Hipperson, John K. Colbourne, Roger C. Dickey, Sam B. Weber, S. James Reynolds

**Affiliations:** 1 School of Biosciences, College of Life and Environmental Sciences, University of Birmingham, Birmingham, UK; 2 NERC Biomolecular Analysis Facility, Department of Animal and Plant Sciences, University of Sheffield, Sheffield, UK; 3 Department of Animal and Agriculture, Hartpury University, Gloucester, UK; 4 School of Geography, Earth and Environmental Sciences, College of Life and Environmental Sciences, University of Birmingham, Birmingham, UK; 5 The Army Ornithological Society (AOS), c/o Prince Consort Library, Knollys Road, Aldershot, Hampshire UK; 6 Ascension Island Government Conservation and Fisheries Department (AIGCFD), Ascension Island, UK; 7 Centre for Ecology and Conservation, University of Exeter, Penryn, UK

**Keywords:** Molecular ecology, Ecological genetics

## Abstract

When and where animals breed can shape the genetic structure and diversity of animal populations. The importance of drivers of genetic diversity is amplified in island populations that tend to have more delineated gene pools compared to continental populations. Studies of relatedness as a function of the spatial distribution of individuals have demonstrated the importance of spatial organisation for individual fitness with outcomes that are conditional on the overall genetic diversity of the population. However, few studies have investigated the impact of breeding timing on genetic structure. We characterise the fine-scale genetic structure of a geographically-isolated population of seabirds. Microsatellite markers provide evidence for largely transient within-breeding season temporal processes and limited spatial processes, affecting genetic structure in an otherwise panmictic population of sooty terns *Onychoprion fuscatus*. Earliest breeders had significantly different genetic structure from the latest breeders. Limited evidence was found for localised spatial structure, with a small number of individuals being more related to their nearest neighbours than the rest of the population. Therefore, population genetic structure is shaped by heterogeneities in collective movement in time and to a lesser extent space, that result in low levels of spatio-temporal genetic structure and the maintenance of genetic diversity.

## Introduction

Population genetic structure is driven by a number of factors, including divergent selection, genetic drift and mutation^1^ with a null model of a lack of genetic differentiation or panmixia arising from random gene flow^2^. Although complete panmixia is rare, genetic differentiation is low in species with high dispersal capabilities, such as birds^2^, flying insects^3^ and fish^4^. In contrast, philopatric behaviour, and thus reduced dispersal, promotes inbreeding, which may result in genetic differentiation^5^. For geographically isolated populations, genetic diversity is often lower than in mainland populations^6^. For example, mainland populations of Kentish plovers *Charadrius alexandrinus* have high levels of gene flow and genetic panmixia, whereas those breeding on islands have lower genetic diversity, and genetic differentiation from the mainland populations increases with increasing distance from the mainland^7^.

In addition to philopatry, genetic diversity may be affected by other factors such as land barriers and separation during the non-breeding season^8^. Natal site fidelity over many generations may lead to kin groups^9^, with benefits of nesting near genetic relatives including reduced aggression and increased predator vigilance^10^. Fine-scale spatial genetic structure or isolation by distance (i.e. where similarities among genotypes decay with increasing distance^11^) has been recorded in a number of taxa, including mammals^12^, birds^13^, and fish^14^.

Isolation over time may also result in heterogeneity in genetic structure^15^, whereby temporal barriers impede gene flow^16^, giving rise to differentiation within a species occupying the same spatial location. For such a scenario to influence a population’s genetic structure, variation in individual breeding timing must have some heritable basis^17^. In such populations early breeders are more likely to breed with other early breeders as are later breeders with other later breeders. Research has shown that breeding timing often has an additive genetic component (see review by Hendry and Day^15^), and thus the resulting offspring of such early and late pairings are more likely to breed at the same time as their parents. Therefore, ‘dispersal’ between reproductive times will likely decrease with increasing time (much like spatial dispersal in isolation by distance, where dispersal in space increases with increasing distance), giving rise to higher genetic similarity between individuals breeding at similar times and greater differentiation between those breeding at different times. For example, spawning time in rainbow smelt *Osmerus mordax* led to genetic differentiation between early and late spawners to the same stream in eastern Canada^18^. Isolation by time can also operate regardless of spatial location such as in sockeye salmon *Oncorhynchus nerka* that bred at the same time but at different locations (in streams 20 km apart) that were more genetically similar than those individuals breeding in the same stream but at different times (i.e. 13–15 days apart)^19^. Temporal genetic impacts on reproduction have been reported in flowering plants^17^, invertebrates^20^, fish^16^ and birds^21^, including seabirds^22^. However, temporal effects on genetic structure are not common in the literature, especially in respect to within-breeding season effects^23^.

Seabirds are an ideal study system to investigate fine-scale genetic structure given that many species exhibit philopatry^24^, despite their high dispersal capabilities. In fact, seabird populations show highly variable levels of genetic structure. In a meta-analysis, Friesen, et al.^8^ found evidence of genetic structure, at various geographical scales, in 40% of 53 species of seabirds. Previous research on seabirds has considered broad-scale variation in population genetics among geographically-isolated nesting colonies^2,25^. The usefulness of exploring within-population genetic mixing mechanisms for understanding species’ global gene flow and population dynamics was highlighted by Cristofari, et al.^26^, who suggested that basic genetic features and processes may be obscured when analyses focus on larger geographic scales. Although their study did not consider temporal effects, they provided evidence for the importance of fine-scale spatial genetic heterogeneity, driven by variations in habitat quality in a panmictic king penguin *Aptenodytes patagonicus* colony in the Crozet Archipelago in the Southern Indian Ocean.

Sooty terns *Onychoprion fuscatus* are one of the most numerous and globally distributed seabirds, occurring throughout the tropical oceans. However, little is known about the genetic structure of their breeding populations (but see Avise, et al.^27^). Like many pelagic seabird species^28^, sooty terns are in decline and a recent urgent call for the reassessment of their conservation status has been made^29^. Factors such as declining fish stocks, climate change, pollution and introduced predators at breeding grounds^30^ are thought to have contributed to such declines. We studied the population that breeds on Ascension Island in the South Atlantic which represents 40% of Atlantic sooty terns^31^; the Ascension population has declined by 84% from > 2 million to ~ 500,000 individuals between 1942 and 2005, respectively^29^. To investigate genetic structure within the Ascension Island sooty tern population we use microsatellite markers to address three questions: (1) What is the current level of genetic diversity and structure exhibited by the population? (2) Does the population show spatial heterogeneity in genetic structure at different spatial scales? (3) Is there evidence to support within-breeding season temporal genetic differentiation?

Given the recent declines in the Ascension Island population of sooty terns, a within-population approach will aid our understanding of local population dynamics, as well as genetic diversity and potential plasticity to environmental change. To the best of our knowledge, this study is the first to combine within-population genetic, spatial and temporal data to understand population genetic structure of a colonial seabird.

## Materials and methods

### Study site and data collection

The study took place between 25th October 2015 and 26th January 2016 on Ascension, a 97 km^2^ island in the South Atlantic (7° 56ʹ S, 14° 22ʹ W). Sooty terns are small, long-lived colonially nesting seabirds that typically lay one egg per breeding attempt and have long maturation times of approximately 5 years^31^. During incubation both parents incubate the egg and once hatched the chick is guarded for a few days before being left alone to allow both parents to forage at sea^31^. Sooty terns are surface feeders having poor waterproofing and rely on larger marine predators, such as tuna *Thunnus* spp. to drive small fish to the surface where terns feed on them in so-called ‘facilitated foraging’^32^.

**Figure 1:**
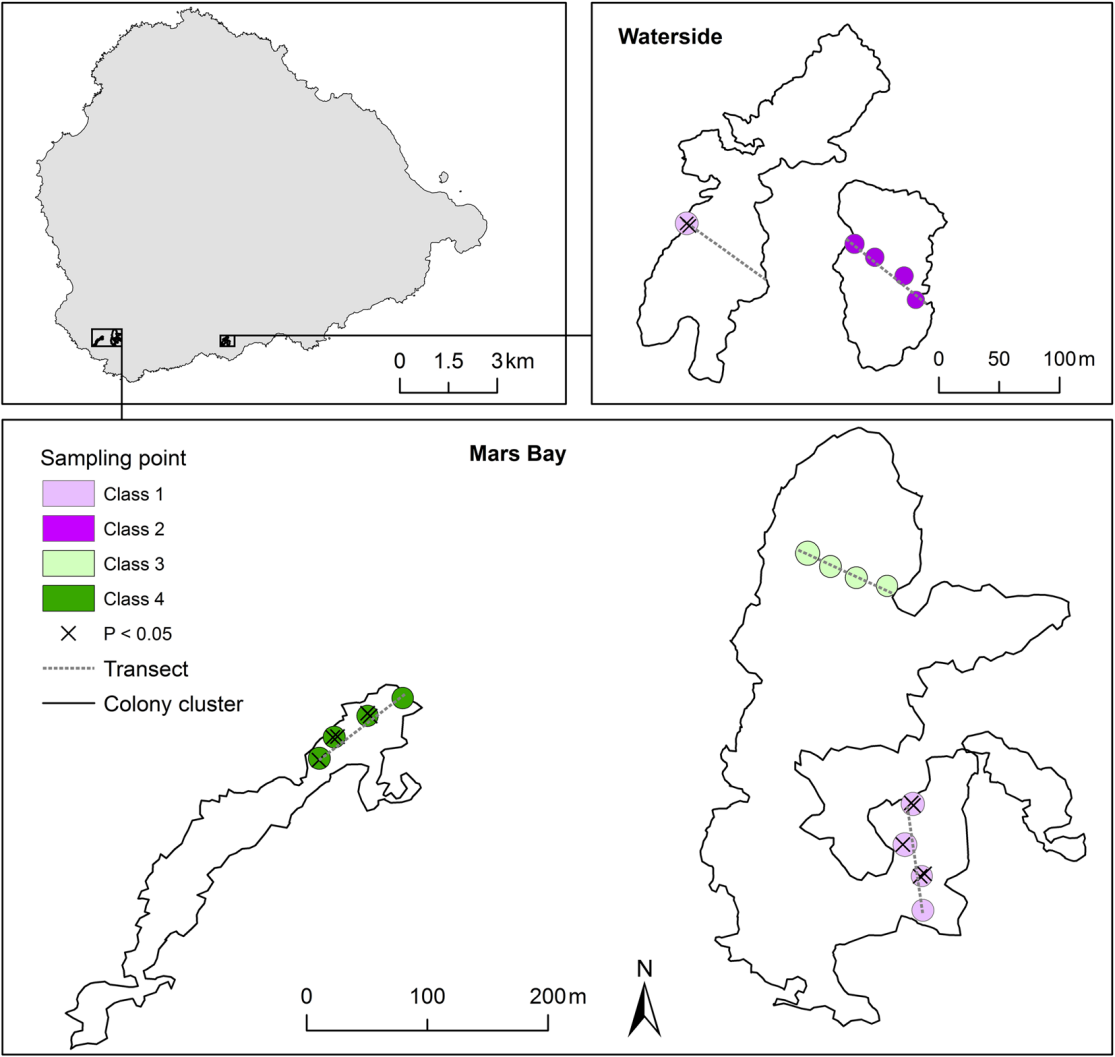
Locations of breeding grounds, nearest neighbour relatedness and breeding timing classes of sooty terns on Ascension Island. Inset map of Ascension Island (top left) shows the locations of the two sooty tern breeding grounds at Mars Bay and Waterside. The locations of 12 individuals significantly more genetically related to their four nearest neighbours than those selected at random from the population are shown within sampling points (marked with Xs). Sampling points are coloured by breeding timing classes with Class 1 being the earliest breeders and Class 4 the latest.

Ashmole^33^ noted that individuals frequented nesting grounds at night before egg laying commenced, and would largely nest in the same location as these preliminary gatherings. Sooty terns on Ascension Island nest in spatially distinct colony clusters at two main breeding grounds: Mars Bay and Waterside (Fig. 1). We estimated population size at breeding grounds from breeding density and colony cluster area (see Hughes et al.^34^ for further details), to give an estimate of population size at each site of: 127,764 ± 16,638 pairs (Mars Bay) and 66,315 ± 4929 pairs (Waterside) (± 95% confidence intervals).

To assess spatial heterogeneity in genotypes, we monitored breeding individuals along five 90 m transects placed at random within colony clusters, across both breeding grounds (Fig. 1). Temporal variation in genetic structure was assessed by taking blood samples from breeders who initiated nests over a 42 day time period. We sampled only late-incubating individuals to reduce the risk of abandonment. Each transect had four sampling points at 0, 30, 60 and 90 m from the edge of the cluster. Where possible, we monitored the nearest 15 nests to each sampling point, totalling 255 nests. We monitored 17 sampling points in total with one transect providing a single sampling point before eggs of birds in the remaining sampling points hatched, and thus, we did not sample adults from the remaining sampling points. We marked nests with numbered flagging tape tied around a nearby rock or a nest tag hammered into the loose substrate. Sooty terns typically lay one egg^31^ which we marked with a number corresponding to that of the nest using a non-toxic permanent marker as a back-up should the nest label have been lost. We captured adult birds using a telescopically handled fish-landing net and ringed them with uniquely numbered British Trust for Ornithology (BTO) metal rings. We also marked birds with a non-toxic permanent marker on one side of the breast (which remained visible at a distance of 5 m for up to four weeks), preventing recapture of the same individuals. We recorded the location of each sampling point using a hand-held GPS unit (eTrex, Garmin, Hampshire, UK) accurate to ± 5 m. To increase nest location precision, we manually recorded each nest on a map and measured the triangular distances between each nest in relation to the sampling point. These were later uploaded to ArcGIS 10.2^35^ to obtain each nest’s spatial coordinates. To estimate hatching dates, we visited nests every three to six days. We collected blood samples from 287 birds (including 77 pairs) at 210 nests. Approximately 100 µL of blood was taken by brachial venepuncture of each bird using a 27G needle and syringe, and stored in 1 mL of 70% ethanol. We made ad-hoc recoveries of recently deceased chicks and stored them at − 20 °C (n = 7). We extracted DNA samples from chick brain tissue.

All experiments were performed in accordance with relevant guidelines and regulations. Blood sampling took place following approval for overseas fieldwork from the local review process of the Animal Welfare Ethical Review Board (AWERB) of the University of Birmingham, UK, and under an Environmental Research Permit issued by the Ascension Island Government (AIG) (ERP-2015-13). The ringing and marking of birds was carried out under a UK BTO ringing licence held by LJHG (permit no. 6316). None of the sampled birds abandoned breeding attempts during the study.

### Genotyping

Genotyping used 26 highly polymorphic autosomal microsatellite markers^36^ together with three sex-typing markers^37,38^. We used the same protocols as those described by Garrett et al.^36^ to extract DNA from blood and tissue samples, conduct PCRs, and amplify and genotype samples.

### Statistical analysis

#### Population genetic diversity and structure

To test whether observed genotypic and allelic frequencies within the population differ from expected frequencies, we assessed deviations from Hardy–Weinberg Equilibrium (HWE) and evidence of Linkage Disequilibrium (LD) in GENEPOP v4.2^39^ using unrelated individuals^40^. We selected non-relatives using the program Friends and Family v21^41^ with relatedness set to < 0.24 (n = 219), assuming a half-sibling relatedness of 0.25. Relatedness values range from − 1 to + 1, with negative values between two individuals suggesting that they are less related on average than two randomly selected birds from the population; positive values represent pairs that are more related than random pairs^41^. To correct for multiple tests, we applied a false discovery rate control^42^ to LD and HWE *P*-values. We assessed observed and expected heterozygosities using CERVUS v3.0.7^43^, which we used as an indication of population genetic diversity^44^. All markers tested were found to be in HWE except *Ofu06*, which displayed an excess of homozygotes. It also displayed a high estimated null allele frequency > 10% (Supplementary Table 1). Therefore, this marker was excluded from subsequent analyses, giving a total of 25 markers. We assessed genotyping error rate using two independent genotypes from the same sample^45^ with 50 individuals re-amplified from extracted DNA and genotyping error rate estimated using the program PEDANT v1.0^46^. Missing alleles across all individuals for the 25 loci amounted to 0.32% with no one individual having more than 15% missing data (see Supplementary Table 1). Therefore, we included all individuals in the analysis.

We tested population-level variation in genetic structure using the program STRUCTURE v 2.3.4^47^. We assigned clusters of individuals with similar variation to one of the n populations (*K*) identified using a burn-in of 100,000 iterations and 500,000 MCMC (Markov chain Monte Carlo) steps with values of *K* from 1 to 10. Ten independent runs per *K* were performed to check for consistency across runs. The likelihood of different values of *K* was then assessed using posterior probabilities. To estimate this variation in the posterior probability, each value of *K* was assessed through 10 iterations. We also used the Evanno, et al.^48^ method to calculate the most likely value of *K* (for *K* > 1) using Structure Harvester v 0.6.94^49^.

We used pairwise relatedness and individual inbreeding coefficients as measures of fine-scale genetic structure in subsequent analyses. We assessed the performance of four relatedness estimators with the R package ‘related’^50^ using all individuals including chicks (n = 294). Wang’s estimator of relatedness^51^ had the best correlation coefficient and was used in subsequent analyses (Pearson’s correlation coefficients using: Li et al. = 0.9364; Lynch and Ritland = 0.8628, Queller and Goodnight = 0.9367; and Wang = 0.9375). All of the chicks genotyped (n = 7) were correctly identified as offspring of their expected parents except for one which was unrelated to its expected paternal parent, suggesting extra-pair paternity. We only analysed chick DNA to assess relatedness estimators and check the reliability of the markers; they were excluded from all other analyses. We estimated individual inbreeding coefficients using Ritland’s method-of-moments estimator (MME)^52^ in the R package ‘related’. We visualised observed inbreeding per individual by mapping a graduated colour ramp onto the colony using ArcGIS v10.2^35^.

### Spatial genetic structure

In populations with some degree of kin-based philopatry and reduced dispersal, there may be evidence of genotypic spatial clustering. Therefore, we assessed genetic structure at various spatial scales. At the wider landscape scale, we calculated genetic differentiation between breeding grounds in GENALEX v 6.5^53^ to estimate the pairwise G″_ST_ with the number of permutations and bootstraps set to 999. We used the G statistic, given the commonly used F_ST_ can have limitations such as a reduced value when heterozygosity is high^54^ when using highly variable loci. To look for evidence of genetic isolation by distance, we performed spatial autocorrelation analysis in GENALEX v 6.5^55^ on all adult genotypes together with their spatial location. We used even distances up to 100 m and a separate test for all distances with even sample sizes (37 distances, Supplementary Fig. 1). We determined correlogram significance using the non-parametric heterogeneity test of Smouse et al.^56^ where the null hypothesis of a non-random distribution of genotypes in space is accepted when *P* < 0.01. We also analysed each sex separately to test for sex-biased dispersal, with comparisons between sexes following Banks and Peakall^57^.

We conducted heterogeneity tests in relatedness at transect- and sampling point-scales using permutations to detect within- and between-transect and sampling point differences. These permutation tests use the same number of individuals from within, compared to between, sampling points, the latter of which were selected at random using 10,000 simulations to obtain the *P* value (see Jacob et al.^58^). We analysed fine-scale genetic spatial structure using the two-dimensional local spatial autocorrelation (2D LSA) statistics from the program GENALEX v 6.5^59^. Given the four nearest individuals are more likely to be an individual’s direct neighbours surrounding a focal nest (LJHG pers. obs.), we set the number of neighbours to four. We inferred local autocorrelation (*lr*) where *P* < 0.05 using permutation tests (100,000 simulations) to calculate significance. We mapped locations of significant *lr* results onto the colony using ArcGIS v10.2.

### Temporal genetic structure

To assess within-breeding season temporal partitioning in genetic structure, we estimated breeding timing using hatching dates calculated as the number of days from the first reported hatching date (i.e. 24th September 2015) (AIGCFD pers. comm.), as laying dates were not always known. We considered evidence for a relationship between genetic structure and breeding timing by assigning timing classes and as a linear relationship. For the former, breeding pairs with known hatching dates (n = 203) were assigned a breeding timing class using a k means clustering algorithm in R^60^, resulting in four such clusters (mean ± SD: Class 1 = 74 ± 1, Class 2 = 82 ± 2, Class 3 = 91 ± 3, Class 4 = 108 ± 3 days from first hatch). We compared genetic differentiation between breeding timing classes using G″_ST_ statistics in GENALEX v6.5 with permutations and bootstraps set to 999. We mapped breeding timing classes onto the colony using ArcGIS v10.2. Inbreeding coefficients of individual birds were linearly regressed against hatching dates that had been Tukey transformed using the R package ‘rcompanion’^61^. We also compared pairwise relatedness with the absolute difference (in days) between pairwise hatching dates using a Mantel test in GENALEX v6.5.

## Results

### Genetic diversity and population structure

**Figure 2:**
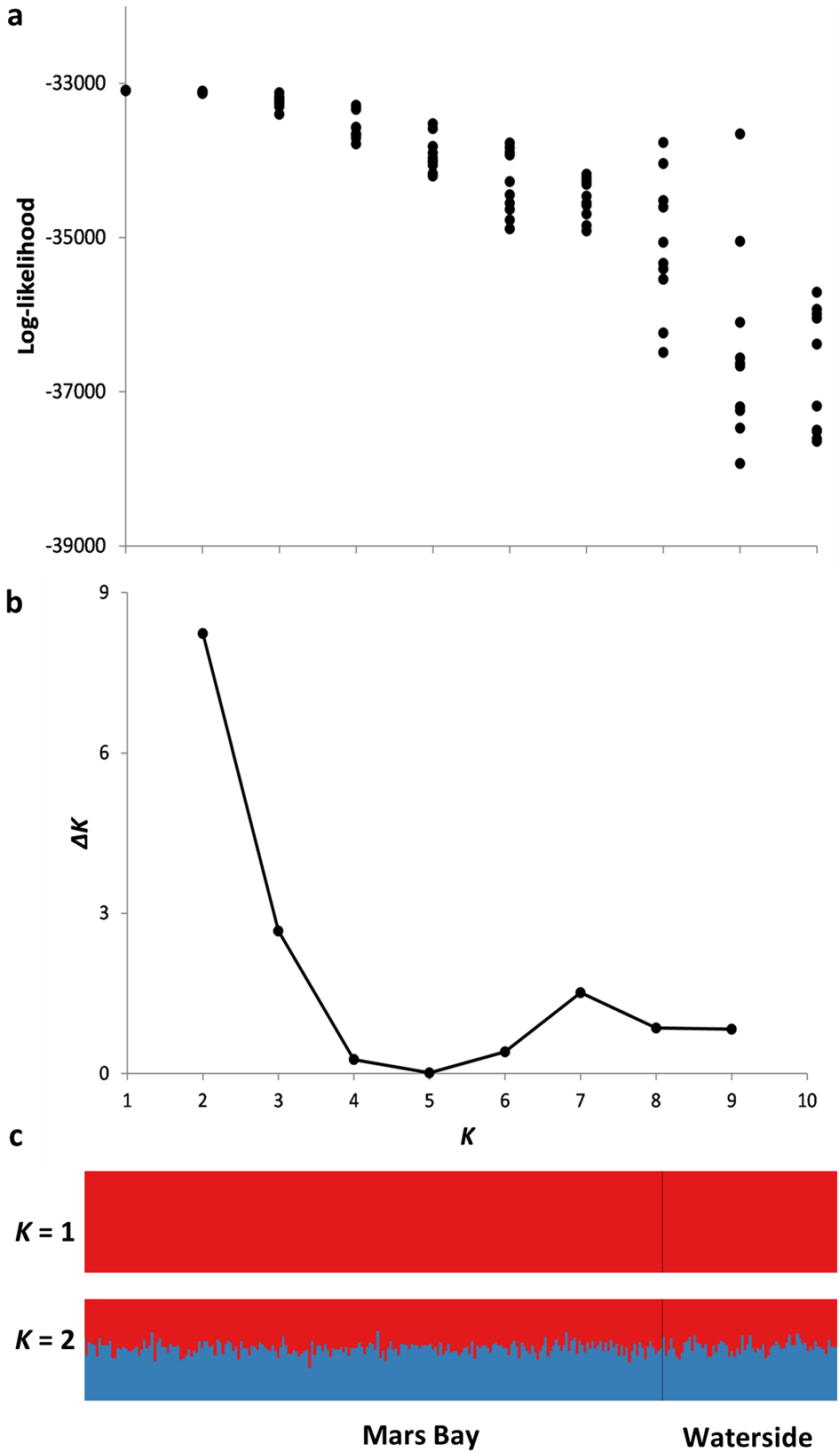
The likelihood of the number of populations (*K*) based on genetic structure using (**a**) the individual log-likelihood values per run (10 runs per *K*) for each value of *K* showing convergence at *K* = 1. (**b**) the most probable number of genetic clusters (*K*) evaluated by the Evanno et al.^48^ method using Δ*K* based on the rate of change in the log-probability of data between successive *K* values. (**c**) genetic structure plots of sooty terns genotyped using 25 microsatellite markers from the two breeding grounds at Mars Bay (n = 217) and Waterside (n = 70), on Ascension Island for *K* = 1 and *K* = 2.

The sooty tern population on Ascension Island has high genetic diversity, with mean observed and expected heterozygosities of 0.80 ± 0.09 and 0.82 ± 0.09, respectively (Supplementary Table 1). The 25 microsatellite loci also had a high average number of alleles (17.36 ± 8.07 alleles per locus, range = 6–38). There was no evidence of population genetic structure identified in STRUCTURE (*K* = 1 had the highest log-likelihood, Fig. 2a) and overall average pairwise relatedness was low (mean = − 0.007 ± 0.086, n = 43,071 pairs). The ΔK analysis also showed steep declines in the number of probable populations at *K* > 2 (Fig. 2b). STRUCTURE analysis indicated that all individuals were evenly split between populations from when *K* was > 1 (Fig. 2c). Individual inbreeding coefficients were also relatively low (mean = 0.006 ± 0.051, range: − 0.09 to 0.23) and showed no obvious clustering when mapped onto the colony (Supplementary Fig. 2).

### Spatial genetic structure

**Figure 3:**
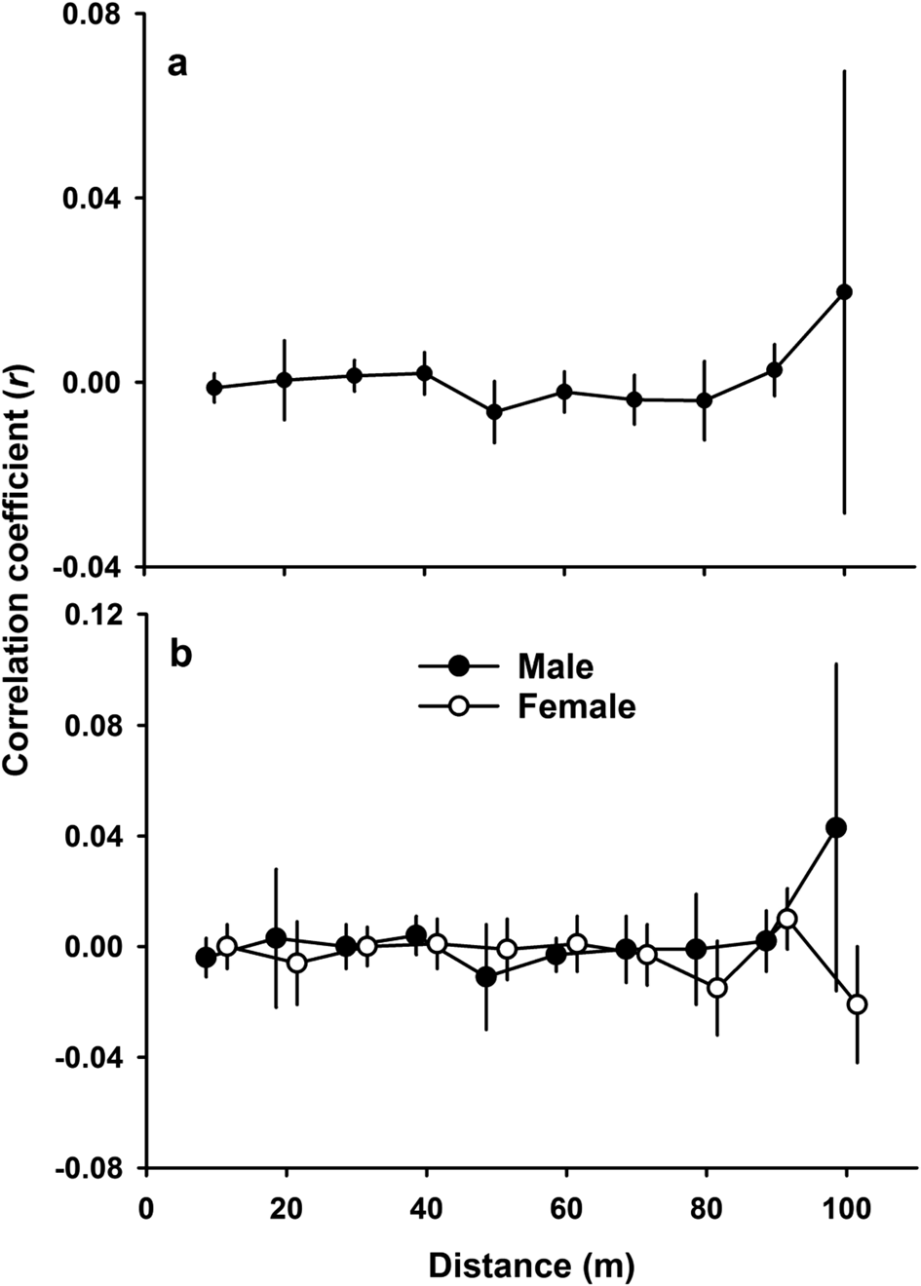
Genetic spatial autocorrelation analysis of sooty terns on Ascension Island (**a**) Population-level spatial correlogram. Solid line: Observed correlation coefficient (*r*) for each distance class, error bars: 95% confidence intervals determined by bootstrapping. (**b**) Spatial correlogram displaying each sex separately. Error bars: 95% confidence intervals determined by bootstrapping.

We found varying degrees of genotypic structuring at different spatial scales and some support for highly localised spatial genetic structure. There was no evidence of landscape-scale partitioning acting on the genetic structure of the population between the two main breeding grounds (G″_ST_ = 0.004, *P* = 0.89). Nor was there any distance-based structuring from autocorrelation analysis, which detects isolation by distance using an alpha of < 0.01 (Omega = 28.1, *P* = 0.02, Fig. 3a, Supplementary Fig. 1), including between sexes (Omega = 12, *P* = 0.90, Fig. 3b, Supplementary Fig. 2). Heterogeneity tests of relatedness either within (*r* = − 0.007 ± 0.086) or between transects (*r* = − 0.007 ± 0.086) showed no significant difference (*P* = 0.55). However, individuals located within sampling points were significantly more related to one another (*r* = − 0.004 ± 0.090) than to individuals at other sampling points (*r* = − 0.007 ± 0.086, *P* = 0.03). There was some evidence of very fine-scale genetic structuring between a small number of neighbouring individuals, with the 2D LSA analysis yielding 12 individuals that were more related to their four nearest neighbours than those selected at random from the population (*r* ranged from 0.07 to 0.13, *P* < 0.05). These individuals were located within three sampling transects (Fig. 1).

### Temporal genetic structure

There was within-breeding season temporal genetic partitioning at the population level. Pairwise comparisons of genetic similarity between breeding timing classes found significant differences only between the earliest and latest timing classes with 34 days between average hatching dates (G_ST_ = 0.012, *P* = 0.017, Table 1). Interestingly, these breeding timing classes were located within the same three transects as the locations of individuals, which were more related to their four nearest neighbours (Fig. 1). However, there was no support for temporal genetic effects as a linear association (breeding timing and individual inbreeding coefficients: *F*_1, 201_ = 1.41, *R*^2^ = 0.002, *P* = 0.24; pairwise relatedness estimates and absolute differences in breeding timing: Mantel *R*^2^ = 0.0002, *P* = 0.24).

**Table 1:** Pairwise comparisons of sooty tern genetic differentiation between breeding timing classes on Ascension Island.

	Class 1	Class 2	Class 3	Class 4
Class 1		-0.011	− 0.005	0.012
		0.916	0.781	0.017*
		8	17	34
Class 2			− 0.012	0.002
			0.923	0.432
			9	26
Class 3				0.007
				0.092
				17
Class 4				

Breeding pairs with known hatching dates (n = 203) were assigned a breeding timing class using a k means clustering algorithm, resulting in four clusters (Class 1: n = 53, Class 2: n = 27, Class 3: n = 58, Class 4: n = 65).

Genetic differentiation is displayed using G″_ST_. The G″_ST_ coefficient is shown followed by the *P* value and the number of days between average hatch times. Significant differences between classes are shown in bold text with an asterisk.

## Discussion

We found temporal genetic structure and limited evidence for very local scale spatial genetic structure in an otherwise highly genetically diverse population. We found evidence for the importance of within-breeding season timing effects on genetic structure which parallels those found in other organisms, such as migratory fish species^15,18^. The observed overall heterogeneity in temporal, and, to a lesser extent, spatial effects on genetic structure, suggests a trade-off between these mechanisms. Together with processes such as dispersal between populations, temporal partitioning, and very low levels of spatial synchrony, may have resulted in genetic diversity being maintained within the population.

### Population panmixia

High population genetic diversity, coupled with a lack of genetic differentiation between breeding colonies (i.e. Mars Bay and Waterside), suggests that landscape-scale barriers to gene flow are not operational on the island. There was also no evidence of genetic isolation by distance (Fig. 3a) or sex-biased dispersal (Fig. 3b). Together with low levels of relatedness and inbreeding, this suggests within-population breeding dispersal. Given seabirds are long-lived species with high dispersal capabilities, there is also the potential for between-population gene flow^62^. Non-breeding distributions of seabirds have been highlighted as key predictors of genetic structure and gene flow^8^, with those staying close to their breeding grounds displaying greater genetic structure. In terns (Sternidae), philopatry usually occurs once breeders have undertaken their first breeding attempts^63^, with natal dispersal often considerably exceeding adult breeding dispersal^64^. Pre-breeder dispersal in Leach’s storm-petrels *Oceanodroma leucorhoa* demonstrates such a mechanism for genetic mixing between subpopulations and the persistence of geographically isolated populations despite increased rates of predation^65^. Evidence of gene flow into the Ascension Island sooty tern population includes: (i) a retrap of a bird ringed as a juvenile off the coast of Brazil that subsequently bred on Ascension Island^66^, (ii) a pre-breeding bird originally from Ascension Island recovered in Abidjan in Côte d’Ivoire, West Africa, and (iii) recoveries of juvenile birds from the Dry Tortugas, FL, USA that were made in the Gulf of Guinea off West Africa^67^. Therefore, it is likely that before breeding for the first time, birds fledged from Ascension Island and the Dry Tortugas may forage together in the Gulf of Guinea. Preliminary genetic analyses on the differentiation between populations also revealed similarities within but not between ocean basins, with differences observed between the Atlantic and Indo-Pacific populations^27^. The relatively recent global scale population expansion, within the last 100,000 years, is also likely to influence contemporary genetic diversity^68^. Thus, inter-population dispersal could be a key process that maintains genetic diversity within the Ascension Island population and indeed within the species as a whole.

### Fine-scale spatial genetic structure

At finer scales there was some limited evidence of genetic structuring. Individuals within sampling points (within 5 m of each other) were more related to one another than those located at other sampling points. Localised spatial structure was also evident in 12 individuals that were more related to their nearest neighbours than to the rest of the population (Fig. 1). The synchronised movement of groups of individuals in space is likely to evolve where conflicts between group members are low relative to group cohesion benefits^69^. The benefits of nesting near genetic relatives include reduced aggression and increased vigilance against predators^10^, reduced nest site competition^70^, infanticide avoidance^71^ and familiarity with an area with respect to habitat features and risks from predators^72^.

### Temporal genetic structure

There was evidence of genetic structuring related to intragroup breeding synchrony with earliest breeders having significantly different genetic structure from the latest breeders (i.e. birds breeding > 1 month later, Table 1). Coordinated group movement in time is likely to evolve where the conflicts between group members are relatively high (although not higher than group cohesion benefits^69^). For example, if group members differ in their previous breeding season success rates, their optimum return times for the following season will also differ, given higher investment in offspring by successfully breeding adults. An extreme example of this is black-browed albatrosses *Thalassarche melanophris* that breed at South Georgia in the Southern Atlantic Ocean. They delay breeding by up to four years following successful fledging of young, whereas around half of breeders failing during incubation return to breed the following year^73^. Competition for nest sites was also thought to be a driving factor in the genetic divergence of two breeding populations of band-rumped storm petrels *Oceanodroma castro* that breed on the same colony in the Galápagos Islands but at different times of the year (6 months apart)^74^. Timing-related genetic mechanisms show high heritability^16,75^ and may arise due to high competition for resources such as space, food and mates^23^. Indeed, Casagrande et al.^76^ suggested that the relatively high genetic differentiation between early and late breeding Eurasian kestrels *Falco tinnunculus,* breeding one month apart in close proximity, may be a result of variable food supply and weather conditions. Differences in population-level genetic structure according to timing of breeding have also been observed in a salmonid fish *Oncorhynchus* spp. with differences in arrival times of as little as 2 weeks apart^15^, although, few studies have investigated within-breeding season effects on genetic structure in birds (but see Casagrande et al.^76^).

### Heterogeneity in spatial and temporal synchronies

Spatial and temporal synchronies are not always independent^16^ and the earliest and latest breeding birds in our study matched spatially with those that showed higher genetic relatedness to their nearest neighbours (Fig. 1). However, a trade-off in collective movement between space and time may also occur, where individual benefits are disparate^69^. For example, an individual’s optimum breeding time may be different from that of the group in which it is contained. This is likely as sooty terns have a low rate of re-nesting if the initial breeding attempt fails^33^, because of the large investment of energy and time in each breeding attempt. Thus, heterogeneity in breeding success in both space and time may give rise to transient movement behaviours, where genetic mixing occurs across a gradient, resulting in only local scale genetic structuring.

Information exchange between individuals can also be important in determining a species’ spatial and temporal resource use^77^. Local reproductive outcome rather than individual breeding success has been shown to influence whether returning breeders maintain group cohesion in the future in colonially nesting black-legged kittiwakes *Rissa tridactyla*^78^. Thus, information about group-level breeding success may determine with whom individuals nest the following season. Francesiaz, et al.^79^ found social connections in slender-billed gulls *Chroicocephalus genei* were maintained temporally, despite breeding site fidelity being low, with colony fidelity dependent on breeding success in the previous season.

### Genetic adaptability

**Figure 4:**
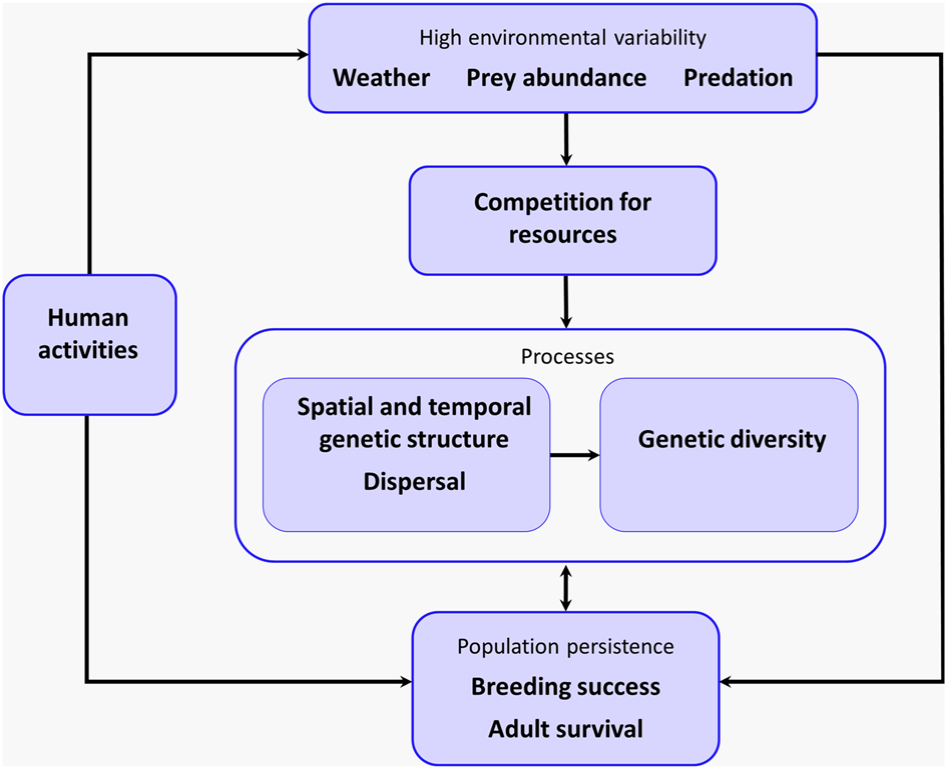
Potential factors influencing the evolutionary strategies and population persistence of sooty terns on Ascension Island and their interactions. A highly variable natural environment often leads to increased competition for space and food. Possible adaptive strategies to enable increased genetic plasticity and thus population persistence may include low levels of fine-scale spatial and temporal structure, coupled with dispersal within- and between-populations. Human activities increase the severity of environmental stressors (e.g. through anthropogenic climate change, overfishing and introduced predators) and limit population persistence through direct impacts on breeding success and survival (such as via egg harvesting).

High levels of genetic diversity are thought to enhance species’ recovery after experiencing extreme climatic conditions. For example, Reusch, et al.^80^ found that increased genotypic diversity in eelgrass *Zostera marina* led to higher plant density and biomass production after exposure to near-lethal temperatures. Populations of yellow warblers *Setophaga petechia* with the least genomic variation were also predicted to be most vulnerable to extinction under projected climate change scenarios and indeed are already experiencing population declines^81^. High genetic diversity and thus population persistence is likely to be a result of a combination of multiple factors (Fig. 4). In species subject to high environmental variability, such as extreme weather events, variable prey abundance and predation, competition for both space and food is often high, which has been demonstrated in some seabird species^82^. Possible adaptive strategies to combat such stressors may include low levels of spatial and temporal genetic structure, as well as dispersal between breeding populations, which leads to genetic diversity being maintained (Fig. 4). For example, Dobson et al.^75^ found high heritability of phenotypic variation in breeding timing in common terns *Sterna hirundo* with strong selection for earlier laying having a positive effect on fecundity. However, high levels of phenotypic plasticity enabled adaptation to high sea surface temperature (SST) that negatively affected wintering feeding grounds, resulting in delays in breeding timing and selecting against early laying. However, although high genetic diversity and life-history traits such as longevity promote plasticity in the face of a changeable environment, with single-season breeding failures having little effect on population persistence, recurrent breeding failures and low adult survival due to low food availability, for example, will result in population declines^83^, even with high genetic diversity. Anthropogenic negative impacts on seabirds through overfishing, climate change, introduction of invasive species^84^, egg harvesting^85^ and other disturbance at nesting grounds^86^ increase pressure on such a population that could exceed its adaptive capacity (for example, altering nesting habitat to avoid predators as shown by sooty terns breeding in the Dry Tortugas^87^) (Fig. 4).

## Conclusions

Our findings provide evidence for genetic temporal partitioning and less support for fine-scale spatial genetic structure in an otherwise panmictic seabird population. Local-scale structure could be dependent on factors such as breeding success, information exchange and competition for resources. Heterogeneity in any potential benefits to individuals from collective group structure in time and, to a lesser extent, space may have led to the observed temporal synchrony and low levels of genetic spatial structure, thereby resulting in the observed high genetic diversity within the population. These processes, together with the potential for juvenile dispersal between populations, may lead to gene flow at a scale that mitigates philopatry and avoids inbreeding, thereby increasing the likelihood of population persistence over time through adaptability to environmental change. Future studies investigating population structure would benefit from assessing within-population processes and the inclusion of spatio-temporal parameters to enhance our understanding of a population’s ecology and evolution, and the maintenance of population genetic structure.

## Supplementary information

Supplementary Information 1.

Supplementary Information 2.

## Data Availability

The sooty tern *Onychoprion fuscatus* microsatellite sequences are available from Genbank: https://www.ncbi.nlm.nih.gov/bioproject/PRJEB21955. The genotype of each individual is provided in the Supplementary Information 1.
