# Supplementary material for: Spatio-temporal processes drive fine-scale genetic structure in an otherwise panmictic seabird population
Source: Sci Rep. 2020 Nov 26;10:20725. doi: 10.1038/s41598-020-77517-w (PMC7691516; doi:10.1038/s41598-020-77517-w)
Supplement: Supplementary file 2 — Supplementary Information 2. [file 41598_2020_77517_MOESM2_ESM.docx]

**Spatio-temporal processes drive fine-scale genetic structure in an otherwise panmictic seabird population**

Lucy J.H. Garrett, Julia P. Myatt, Jon P. Sadler, Deborah A. Dawson, Helen Hipperson, John K. Colbourne, Roger C. Dickey, Sam B. Weber, and S. James Reynolds

**Supplementary Table 1 ǀ Details of microsatellite markers used for genotyping sooty terns *Onychoprion fuscatus* on Ascension Island, together with genotyping error rates.**

| Locus | Clone name, ENA accession number/Reference | Multiplex/  Fluoro-Label (F) | *n* | A | Allele size range (bp) | H_O_ | H_E_ | Est null allele Freq. | Ԑ_1_ (n = 50) | Ԑ_2_ (n = 50) |
| --- | --- | --- | --- | --- | --- | --- | --- | --- | --- | --- |
| Ofu01 | Trn17616 | 7/NED | 217 | 20 | 197-243 | 0.86 | 0.88 | 0.012 | 0.006 | 0.011 |
|  | LT903723 |  |  |  |  |  |  |  |  |  |
| Ofu02 | Trn23851 | 2/HEX | 218 | 29 | 189-361 | 0.93 | 0.94 | 0.003 | 0.012 | 0.000 |
|  | LT903724 |  |  |  |  |  |  |  |  |  |
| Ofu03 | Trn25452 | 5/6-FAM | 219 | 16 | 138-176 | 0.80 | 0.84 | 0.030 | 0.007 | 0.019 |
|  | LT903725 |  |  |  |  |  |  |  |  |  |
| Ofu04 | Trn4256 | 5/HEX | 219 | 24 | 128-180 | 0.84 | 0.87 | 0.027 | 0.000 | 0.012 |
|  | LT903726 |  |  |  |  |  |  |  |  |  |
| Ofu05 | Trn171 | 4/6-FAM | 216 | 23 | 87-133 | 0.89 | 0.92 | 0.014 | 0.006 | 0.011 |
|  | LT903727 |  |  |  |  |  |  |  |  |  |
| Ofu06* | Trn352 | 7/HEX | 218 | 13 | 246-282 | 0.73 | 0.86 | 0.09 | - | - |
|  | LT903728 |  |  |  |  |  |  |  |  |  |
| Ofu07 | Trn436 | 4/NED | 219 | 10 | 156-208 | 0.82 | 0.88 | 0.039 | 0.010 | 0.000 |
|  | LT903729 |  |  |  |  |  |  |  |  |  |
| Ofu08 | Trn640 | 1/6-FAM | 219 | 38 | 272-368 | 0.87 | 0.90 | 0.016 | 0.010 | 0.000 |
|  | LT903730 |  |  |  |  |  |  |  |  |  |
| Ofu09 | Trn643 | 6/6-FAM | 219 | 19 | 164-212 | 0.92 | 0.92 | -0.001 | 0.017 | 0.000 |
|  | LT903731 |  |  |  |  |  |  |  |  |  |
| Ofu10 | Trn16824 | 2/NED | 218 | 29 | 130-230 | 0.93 | 0.94 | 0.003 | 0.016 | 0.005 |
|  | LT903732 |  |  |  |  |  |  |  |  |  |
| Ofu11 | Trn13992 | 1/6-FAM | 219 | 16 | 156-208 | 0.56 | 0.62 | 0.061 | 0.032 | 0.000 |
|  | LT903733 |  |  |  |  |  |  |  |  |  |
| Ofu12 | Trn129 | 3/6-FAM | 219 | 13 | 300-334 | 0.76 | 0.78 | 0.013 | 0.000 | 0.006 |
|  | LT903734 |  |  |  |  |  |  |  |  |  |
| Ofu13 | Ofu839 | 6/HEX | 219 | 10 | 133-171 | 0.78 | 0.81 | 0.019 | 0.006 | 0.000 |
|  | LT903735 |  |  |  |  |  |  |  |  |  |
| Ofu14 | Ofu897 | 4/HEX | 214 | 12 | 345-369 | 0.83 | 0.81 | -0.01 | 0.007 | 0.006 |
|  | LT903736 |  |  |  |  |  |  |  |  |  |
| Ofu15 | Trn191 | 2/6-FAM | 218 | 14 | 328-356 | 0.78 | 0.79 | 0.007 | 0.015 | 0.000 |
|  | LT903737 |  |  |  |  |  |  |  |  |  |
| Ofu16 | Trn484 | 6/6-FAM | 219 | 9 | 313-331 | 0.66 | 0.67 | 0.007 | 0.006 | 0.000 |
|  | LT903738 |  |  |  |  |  |  |  |  |  |
| Ofu17 | Trn715 | 5/NED | 218 | 15 | 181-213 | 0.77 | 0.86 | 0.056 | 0.000 | 0.000 |
|  | LT903739 |  |  |  |  |  |  |  |  |  |
| Ofu18 | Trn269 | 1/HEX | 218 | 6 | 296-306 | 0.73 | 0.79 | 0.023 | 0.015 | 0.000 |
|  | LT903740 |  |  |  |  |  |  |  |  |  |
| Ofu19 | Trn15 | 6/6-FAM | 219 | 12 | 94-120 | 0.73 | 0.76 | 0.018 | 0.000 | 0.000 |
|  | LT903741 |  |  |  |  |  |  |  |  |  |
| Ofu20 | Trn551 | 3/HEX | 219 | 21 | 217-287 | 0.87 | 0.89 | 0.014 | 0.026 | 0.000 |
|  | LT903742 |  |  |  |  |  |  |  |  |  |
| Ofu21 | Trn121 | 5/6-FAM | 219 | 35 | 265-337 | 0.96 | 0.96 | -0.002 | 0.017 | 0.012 |
|  | LT903743 |  |  |  |  |  |  |  |  |  |
| Ofu22 | Trn652 | 6/NED | 219 | 9 | 150-168 | 0.74 | 0.74 | -0.004 | 0.011 | 0.000 |
|  | LT903744 |  |  |  |  |  |  |  |  |  |
| Ofu23 | Trn407 | 1/HEX | 218 | 15 | 142-204 | 0.80 | 0.80 | -0.002 | 0.005 | 0.000 |
|  | LT903745 |  |  |  |  |  |  |  |  |  |
| Ofu24 | Trn442 | 3/6-FAM | 219 | 9 | 143-181 | 0.86 | 0.84 | -0.013 | 0.000 | 0.000 |
|  | LT903746 |  |  |  |  |  |  |  |  |  |
| Ofu25 | Trn126 | 5/HEX | 218 | 19 | 222-284 | 0.73 | 0.76 | 0.019 | 0.12 | 0.008 |
|  | LT903747 |  |  |  |  |  |  |  |  |  |
| Ofu26 | Trn825 | 4/6-FAM | 218 | 8 | 186-206 | 0.61 | 0.60 | -0.003 | 0.005 | 0.000 |
|  | LT903748 |  |  |  |  |  |  |  |  |  |
| Z002A | Dawson (2007) | 2/6-FAM | 107M | 1 | 249 (Z) | 0 | - | - | - | - |
|  |  |  | 112F | 2 | 249 (Z) and 252 (W) | 1.00 | - | - |  |  |
| Z002D | Dawson (2007) | 1/6-FAM | 107M | 1 | 127 (Z) | 0 | - | - | - | - |
|  |  |  | 112F | 2 | 122 (W) and 127 (Z) | 1.00 | - | - |  |  |
| Z43B | Dawson et al. (2016) | 7/6-FAM | 107M | 1 | 270 (Z) | 0 | - | - | - | - |
|  |  |  | 112F | 2 | 266 (W) and 270 (Z) | 1.00 | - | - |  |  |

ENA is the European Nucleotide Archive: https://www.ebi.ac.uk/ena/data/view/PRJEB21955, *n* is the number of individuals tested, A is the number of alleles observed, H_O_ is the observed heterozygosity, H_E_ is the expected heterozygosity, Est null allele Freq. is the estimated null allele frequency, M is the number of males and F is the number of females identified using the sex-typing markers (Z002A, Z002B (Dawson 2007) and Z43B (Dawson et al. 2016). Ԑ_1_ is the allelic dropout rate, Ԑ_2_ is the false allele rate, note n = 50 for the estimation of genotyping error rate. * Locus *Ofu06* not included in full analysis.

**
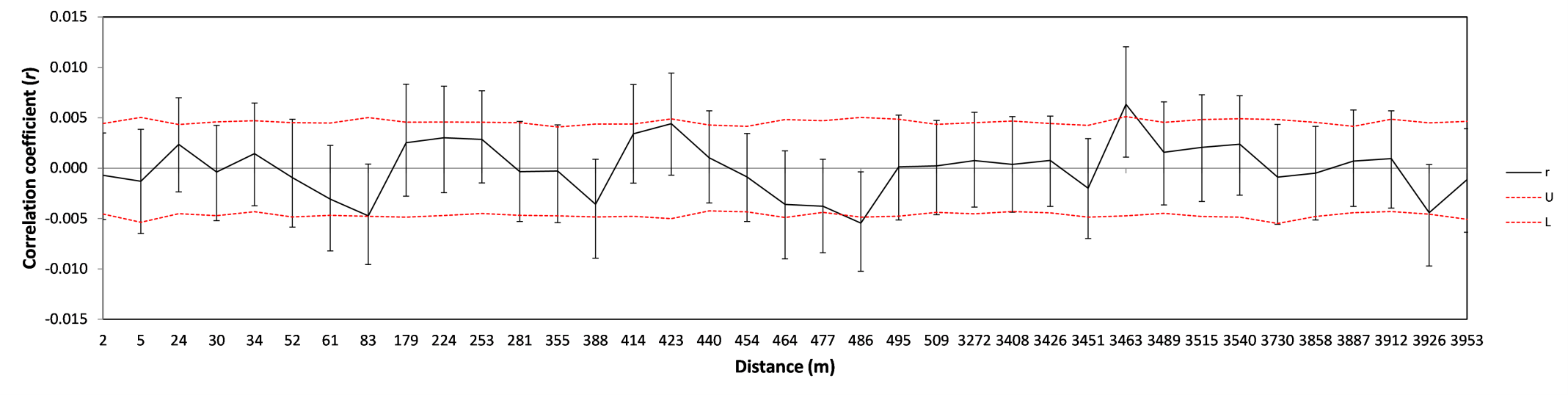
**

**a**

**Correlation coefficient (*r*)**


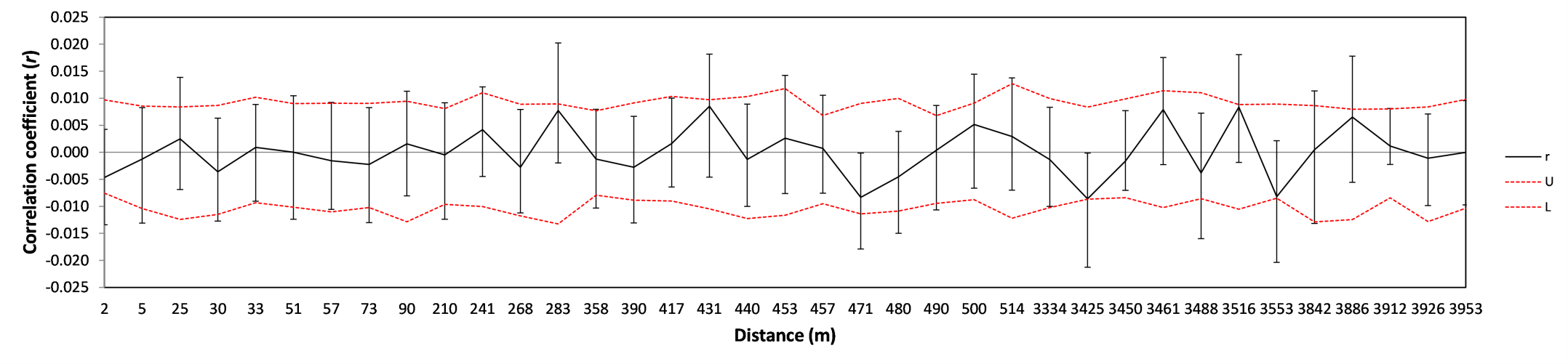


**b**


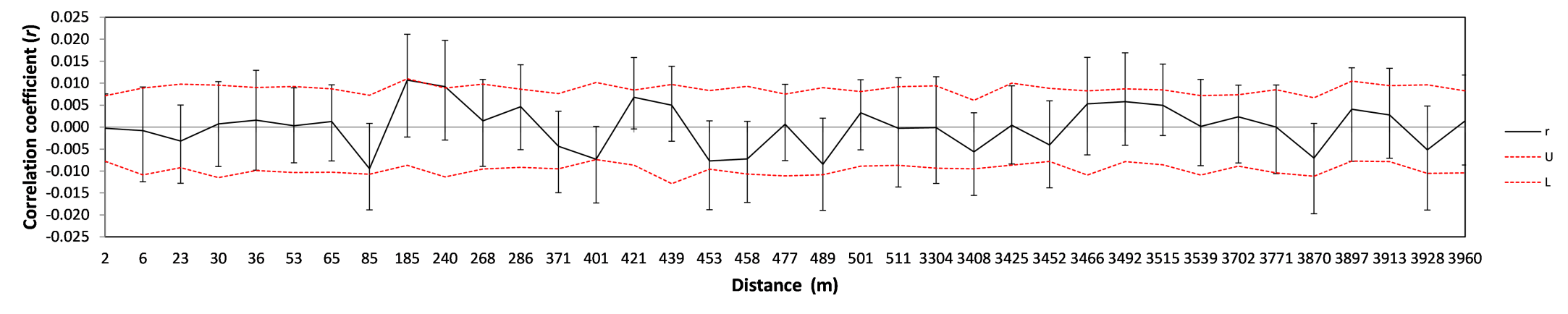


**c**

**Distance (m)**

**Supplementary Figure 1** ǀ **Genetic spatial autocorrelation analysis of sooty terns on Ascension Island with comparisons up to the maximum possible distances between nests a,** at the population level, Omega = 126.83, *P* = 0.02, n sample sizes per distance point range from 1021 to 1395, **b,** for males, Omega = 107.57, *P* = 0.02, n sample sizes per distance point range from 242 to 232 and **c,** for females, Omega = 123.72, *P* = 0.02, n sample sizes per distance point range from 268 to 356. Solid line: Observed correlation coefficient (*r*), Error bars: 95% confidence intervals determined by bootstrapping and Upper (U) and lower (L) confidence limits bound the 95% confidence interval about the null hypothesis of ‘No spatial structure for the combined data set as determined by permutation’.

**
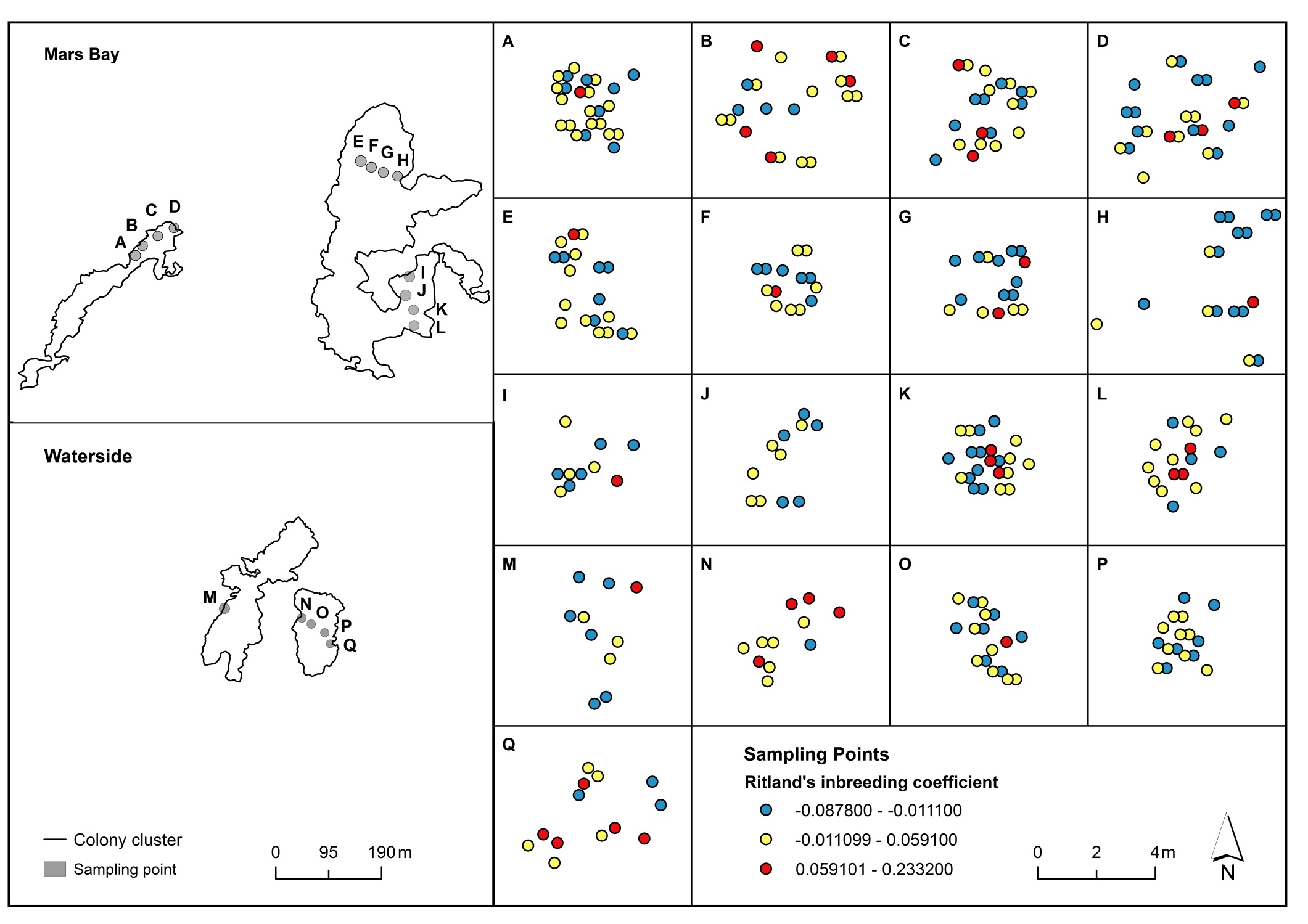
**

**Supplementary Figure 2** ǀ **Spatial distribution of sampling points and individual inbreeding levels across two sooty tern** ***Onychoprion fuscatus* breeding grounds (Mars Bay and Waterside) on Ascension Island.** Left-hand maps show the two breeding grounds separately and the location of sampling points within colony clusters on Ascension Island during peak breeding (Jan 2016). Right-hand boxes depict the spatial distribution of individuals nesting at each sampling point. Coloured points indicate Ritland’s individual inbreeding coefficients. Where both sexes were sampled from the same nest, points have been offset by 30 cm for visualisation purposes.
